# Identification of compounds with activity against *Trypanosoma cruzi* within a collection of synthetic nucleoside analogs

**DOI:** 10.3389/fcimb.2022.1067461

**Published:** 2023-01-13

**Authors:** Berta Barnadas-Carceller, Nieves Martinez-Peinado, Laura Córdoba Gómez, Albert Ros-Lucas, Juan Carlos Gabaldón-Figueira, Juan J. Diaz-Mochon, Joaquim Gascon, Ignacio J. Molina, María José Pineda de las Infantas y Villatoro, Julio Alonso-Padilla

**Affiliations:** ^1^ Barcelona Institute for Global Health (ISGlobal), Hospital Clinic - University of Barcelona, Barcelona, Spain; ^2^ Secció de Parasitologia, Departament de Biologia, Sanitat i Medi Ambient, Facultat de Farmàcia i Ciències de l’Alimentació, Universitat de Barcelona, Barcelona, Spain; ^3^ Department of Medicinal & Organic Chemistry and Excellence Research Unit of “Chemistry Applied to Biomedicine and the Environment”, Faculty of Pharmacy, University of Granada, Granada, Spain; ^4^ CIBER de Enfermedades Infecciosas, Instituto de Salud Carlos III (CIBERINFEC, ISCIII), Madrid, Spain; ^5^ GENYO, Centre for Genomics and Oncological Research, Pfizer/University of Granada/Andalusian Regional Government, PTS Granada, Granada, Spain; ^6^ Biosanitary Research Institute of Granada (ibs.GRANADA), University Hospitals of Granada-University of Granada, Granada, Spain; ^7^ Institute of Biopathology and Regenerative Medicine, Centre for Biomedical Research, University of Granada, Granada, Spain

**Keywords:** Chagas disease, *Trypanosoma cruzi*, purine derivates, antiparasitic assays, cytotoxicity assays, drug discovery cascade

## Abstract

**Introduction:**

Chagas disease is caused by the protozoan parasite *Trypanosoma cruzi*, and it is the most important neglected tropical disease in the Americas. Two drugs are available to treat the infection, but their efficacy in the chronic stage of the disease, when most cases are diagnosed, is reduced. Their tolerability is also hindered by common adverse effects, making the development of safer and efficacious alternatives a pressing need. *T. cruzi* is unable to synthesize purines de novo, relying on a purine salvage pathway to acquire these from its host, making it an attractive target for the development of new drugs.

**Methods:**

We evaluated the anti-parasitic activity of 23 purine analogs with different substitutions in the complementary chains of their purine rings. We sequentially screened the compounds' capacity to inhibit parasite growth, their toxicity in Vero and HepG2 cells, and their specific capacity to inhibit the development of amastigotes. We then used in-silico docking to identify their likely targets.

**Results:**

Eight compounds showed specific anti-parasitic activity, with IC_50_ values ranging from 2.42 to 8.16 μM. Adenine phosphoribosyl transferase, and hypoxanthine-guanine phosphoribosyl transferase, are their most likely targets.

**Discussion:**

Our results illustrate the potential role of the purine salvage pathway as a target route for the development of alternative treatments against *T. cruzi* infection, highlithing the apparent importance of specific substitutions, like the presence of benzene groups in the C8 position of the purine ring, consistently associated with a high and specific anti-parasitic activity.

## Introduction

Chagas disease, also known as American trypanosomiasis, is a Neglected Tropical Disease (NTD) that affects 6-7 million people worldwide (Lidani et al., 2019; WHO, 2021). It is caused by the hemoflagellate protozoan parasite *Trypanosoma cruzi* (*T. cruzi*), which is mostly transmitted by reduviid insects from the subfamily *Triatominae* (triatomine bugs), endemic to the Americas. Alternative transmission routes include the consumption of parasite contaminated food and water, blood transfusions, organ transplants, and vertical transmission from infected mothers to their newborns, the three latter being also of relevance in non-endemic regions of North America, Europe, Australia, and Japan (Pérez-Molina and Molina, 2018; Lidani et al., 2019).

The initial stage of the infection is usually asymptomatic or courses with mild flu-like symptoms, so it mostly goes undiagnosed and untreated. Following this stage, the infection becomes chronic, and the parasite persists for several years without causing overt clinical signs. However, it is estimated that up to 30% of those chronically infected will develop life-threatening cardiac and/or digestive manifestations such as dilated cardiomyopathy, heart failure, megacolon or megaesophagus (Pérez-Molina and Molina, 2018; Lidani et al., 2019; CDC - Chagas Disease, 2021). As a consequence, Chagas disease is a major public health problem in Latin America, where it is considered the main driver of non-ischemic heart failure, leading to ~10,000 deaths per year (CDC - Chagas Disease, 2021).

There are two antiparasitic drugs - benznidazole (BNZ) and nifurtimox (NFX) - available to treat the infection. Although they are highly efficient during the acute stage of the disease, their efficacy is reduced in the chronic stage, when the majority of patients are diagnosed. Moreover, frequent adverse effects and long administration regimens hinder their acceptability and patients adherence to treatment (Jackson et al., 2010; Aldasoro et al., 2018; Pérez-Molina and Molina, 2018). Thus, the development of new drugs, particularly for the chronic stage of the infection, is urgently needed (Beltran-Hortelano et al., 2022).

In the last decade, drug discovery efforts have been based on the screening of large chemical collections, mostly relying on phenotypic assays (Kratz, 2019; García-Huertas and Cardona-Castro, 2021). Nonetheless, the identification of targets to elucidate compounds´ mechanisms of action will greatly contribute to the rational design for less toxic effects and it is highly desirable (García-Huertas and Cardona-Castro, 2021). Ideally, molecular targets need to be essential for parasite growth or survival, have a *druggable* active site, lack homology with proteins present in humans to anticipate reduced toxicity, and have no isoforms within the same parasite species to avoid the occurrence of resistance. Up to date, several studies on target-based anti-*T. cruzi* drug discovery have been published, mostly related to the ergosterol biosynthesis pathway, the cysteine-peptidase cruzain, the proteosome and the enzyme trypanothione reductase (Santos et al., 2020). Among those, posaconazole and E-1224, a prodrug of ravuconazole, both C14-α-demethylase (CYP51) inhibitors, reached clinical trials but failed as monotherapy and were less effective than BNZ (Santos et al., 2020). Fexinidazole, a 5-nitroimidazole derivative in use for African trypanosomiasis, has been more recently clinically evaluated for Chagas disease in a proof-of-concept study, but safety concerns led to enrollment interruption (Torrico et al., 2022). GNF6702 which is an azabenzoxazole compound that acts as an allosteric proteasome inhibitor, matched efficacy with BNZ in a chronic *in vivo* model and is being evaluated in preclinical toxicity studies (Vermelho et al., 2020). Some oxaborole compounds have also been nominated as clinical candidates for the treatment of Chagas disease. For instance, Padilla et al. reported that the benzoxaborole prodrug AN15368, which targets the mRNA processing pathway in *T. cruzi*, was uniformly curative in non-human primates with long-term naturally acquired infections (Padilla et al., 2022). Besides, the oxaborole DNDI-6148 showed high curative rates in mouse models of infection and is currently being evaluated in phase I clinical trials (De Rycker et al., 2022).

Despite being a major public health problem, the development of new therapies against Chagas disease is hindered by its limited financial incentives, leading to most research being carried out directly in academic institutions. Medicinal chemistry in pair with bioinformatic approaches provide valuable tools to advance in this direction (Santos et al., 2020; García-Huertas and Cardona-Castro, 2021). In this regards, whole genome sequencing of *T. cruzi* and the generation of novel biochemical data have been important in the search for new antiparasitic drugs. A pathway of interest is that of purine metabolism, as it is known to hold remarkable differences between *T. cruzi* and mammals (Berg et al., 2010). Purines are of great importance to all organisms since they are the precursors of fundamental components of DNA and RNA macromolecules, act as second messengers, and constitute several coenzymes. *T. cruzi* parasites are unable to synthesize purines *de novo* since they cannot produce inosine monophosphate (IMP) from glycine. Because of that, they need a purine salvage pathway, a route in which purines are synthetized from endogenous and exogenous substrates (Gutteridge and Gaborak, 1979; Berens et al., 1981). Enzymes involved in the salvage pathway show little homology with its mammal counterparts and have already been identified as potential targets of compounds with specific activity against kinetoplastid parasites, including *T. cruzi.* (Tran et al., 2017; Hulpia et al., 2018; Bouton et al., 2021; Martinez-Peinado et al., 2021b).

To further investigate the antiparasitic role of purine analogs, we screened a collection of 23 synthetic compounds for *in vitro* activity against *T. cruzi*. These compounds are based on a collection previously tested against *T. cruzi* and *P. falciparum*, whose solubility has been improved by increasing their hydrophilicity (Martinez-Peinado et al., 2021b).

## Materials and methods

### Chemical collection

**Figure 1:**
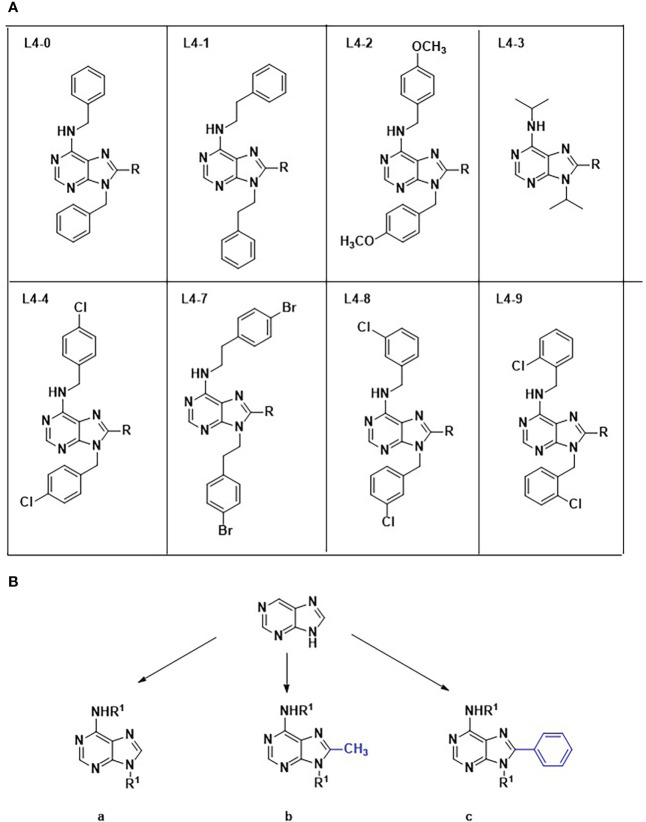
Chemical structures and classification of compounds evaluated in this work. Numbers 0-4 & 7–9 identify the eight different chemical families **(A)**, while their accompanying letters depict scaffold subgroups **(B)**.

The collection comprised twenty-three 6-aminopurine analogs belonging to eight families differentiated by the R1 substituents presented at the exocyclic amino at positions 6 and 9 of the purine ring (L0-L4 & L7-L9). Members of each family present the same R1 at both positions and they were further divided into subtypes a, b or c according to the radical at position 8, which can be H (subfamily a), methyl (subfamily b) and phenyl (subfamily c). All compounds were synthesized from their 4,6-dialky/diarylalkylamino -5-nitropyrimidine derivatives (See Synthesis and characterization of the chemical collection **Supplementary Material** for full details). Briefly, following the reduction of the 5-nitro group with tin (II) chloride the final cyclization steps were carried out with the corresponding trialkylorthoesthers and methanesulfonic to yield the twenty-three members library. All compounds were purified by column chromatography and characterized by nuclear magnetic resonance (1h-NMR, 13C-NMR) and high-resolution mass spectrometry (HRMS). They share a common purine structure, attached to two different functional groups on carbons C6, C8 and nitrogen N9, as shown in **Figure 1**. The absence of a functional group in C8, or the presence of either a methyl or a benzyl group, respectively classified the compounds in scaffold groups a, b, or c. Compounds were provided at a concentration of 100 mM in dimethyl sulfoxide (DMSO) and aliquoted upon arrival. The stock was long term stored at -20°C and daily use aliquots at 4°C. To ensure good solubility of the compounds before running the assays, they were placed in a thermoblock at 40°C for about 10 - 15 minutes in advance of their addition to the assay plates.

### Host cells and *T. cruzi* parasite cultures

Vero (green monkey kidney epithelial cells), LLC-MK2 (rhesus monkey kidney epithelial cells), and HepG2 cells (human liver epithelial cells) were maintained in T75 or T175 flasks with high-glucose/glutamine Dulbecco’s Modified Eagle Medium Phenol Red (DMEM-PR) supplemented with 1% penicillin-streptomycin (PS) and 10% inactivated fetal bovine serum (FBS) (Martinez-Peinado et al., 2020). HepG2 cultures were additionally supplemented with 1× non-essential amino acids solution (NEAA) (Martinez-Peinado et al., 2020).

*T. cruzi* parasites from the Tulahuen strain (DTU VI) expressing β-galactosidase (Buckner et al., 1996) were grown using LLC-MK2 cells as hosts and DMEM-PR supplemented with 2% FBS and 1% PS. Trypomastigotes were isolated for the maintenance of the cycle and for the performance of the antiparasitic assays, respectively. Briefly, medium containing free swimming trypomastigotes was harvested in falcon tubes and centrifuged at 2,500 rpm for 10 minutes to remove cell debris. Supernatant was then carefully aspirated and either replaced with DMEM-PR supplemented with 2% FBS and 1% PS, or with DMEM without PR supplemented with 1% penicillin-streptomycin-glutamine (PSG), 4-2-hydroxyethyl-1-piperazineethanesulfonic acid (HEPES) 25 mM and sodium-pyruvate 1mM in addition to 2% FBS and 1% PS (assay medium) (Martinez-Peinado et al., 2020). In the second case, another centrifugation was made to get rid of any PR remnants that may interfere in the assay readout. Supernatant excess was discarded, and the tubes were incubated at 37°C for at least 1.5 h to allow the trypomastigotes to swim out of the pellet (Buckner et al., 1996).

### *T. cruzi* growth inhibition assay

Anti-*T. cruzi* assays were performed using Vero cells and *T. cruzi* Tulahuen parasites (Martinez-Peinado et al., 2020). Compounds were added to a flat 96-well cell culture plate (SPL Life Sciences, Pocheon-si, Korea) and diluted in assay medium following a 1:2 dose-response pattern. Vero cells were detached, washed and counted using a Neubauer chamber. On the other side, *T. cruzi* trypomastigotes were collected and counted. Then, 50,000 trypomastigotes and 50,000 Vero cells were simultaneously added to each well to reach a final volume of 200 μl per well (multiplicity of infection or MOI = 1). Negative (untreated Vero cells and parasites, indicating 0% inhibition) and positive controls (trypomastigotes alone, indicating 100% inhibition or minimum growth) were included in each run (Martinez-Peinado et al., 2020). Note that trypomastigotes are unable to multiply and therefore they mark the enzymatic time zero. In addition to those controls, BNZ-treated wells were also included as a positive control of drug inhibition. Plates were incubated at 37°C for 96 h. After that, chlorophenol red-β-D-galactopyranoside (CPRG) substrate was added to detect the enzymatic activity of β-galactosidase expressed by the parasites. For this, a PBS dilution with 0.25% of detergent NP-40 and 500 μM of CPRG were added to each well and incubated at 37°C for another 4 h (Bettiol et al., 2009). Absorbance was measured at 590 nm in an Epoch Gene5 Microplate Spectrophotometer (BioTek, Winooski, USA).

### Cytotoxicity assays

Compounds were added to the plates and diluted following a dose-response pattern in assay medium. Vero cells were prepared in assay medium and added to each well as described (Martinez-Peinado et al., 2020). Each test plate contained its own negative (untreated cells) and positive (medium alone) control wells (Martinez-Peinado et al., 2020). BNZ and digitoxin (DTX) were included as non-toxicity and toxicity drug controls, respectively. Parallel assays with HepG2 cells were carried out to evaluate the toxicity of compounds in a human cell line as described (Martinez-Peinado et al., 2020). Plates were incubated at 37°C for 96 (Vero) or 48 hours (HepG2). Vitality was measured with Alamar Blue (Thermo Fischer Scientific, Waltham, USA) to detect metabolically active (live) cells by fluorimetry. For this, a PBS dilution with 10% Alamar Blue was added to each well and the plates were incubated at 37°C for 6 h. Readout was performed on a TECAN Infinite M Nano+ reader (Tecan Trading AG, Männedorf, Switzerland) using i-control™ 2.0 software and setting excitation at 530 nm and emission at 590 nm.

### Amastigotes growth inhibition assay

To address compounds activity against amastigotes, Vero cells were seeded in T-175 flasks (5×10^6^ cells) and cultured for 24 h in DMEM-PR supplemented with 10% FBS and 1% PS. Upon washing the monolayers with PBS, purified trypomastigotes were added at a concentration of 1×10^7^ trypomastigotes per flask (MOI = 1) and infected monolayers were kept in assay medium. After 18 h, infected Vero cells were PBS-washed and trypsin-detached, diluted at a concentration of 5×10^5^ cells per ml in assay medium and 100 μl of that solution (50000 cells) added to 96-well test plates already containing the compounds. Plates were incubated at 37°C for another 72 h and read with CPRG, as described for the anti-*T. cruzi* assay (Martinez-Peinado et al., 2021a; Martinez-Peinado et al., 2021b; Martinez-Peinado et al., 2021c).

### Computational analysis

It was performed to address the potential mechanism behind the effect of compounds specifically inhibiting *T. cruzi* growth. We focused on evaluating the interactions of prioritized compounds with enzymes *Tc*ADSL, *Tc*AK, *Tc*APRT, *Tc*HGPRT, *Tc*IAGNH, *Tc*IGNH, *Tc*IMPDH, and *Tc*MTAP, given their key role in the purine salvage pathway. Computer generated models of these proteins were obtained from the AlphaFold Database (Jumper et al., 2021). Due to the lack of properly annotated protein sequences for the Tulahuen strain, we used protein models of the CL Brener Esmeraldo-like strain of *T. cruzi.* Tridimensional structures of the compounds were created using Avogadro editor software (Hanwell et al., 2012), and the molecular geometry was optimized to obtain the lowest energy values. Natural ligands were obtained from PubChem (Kim et al., 2019) as MOL files, namely adenine, adenosine, adenyl succinate, guanine, guanosine, hypoxanthine, inosine, IMP, SAICAR, and methylthioadenosine. All structures were prepared for docking simulations by adding polar hydrogens and formatting them as PDBQT files using AutoDockTools 1.5.7 (Forli et al., 2016).

Docking was performed with AutoDock Vina 1.1.2 (Eberhardt et al., 2021). The binding box used was sized and centered on the active site of each receptor based on predictions of P2Rank, a prediction algorithm of binding sites based on machine learning, *via* the PrankWeb server (https://prankweb.cz/; Jendele et al., 2019). Exhaustiveness and energy range were set to 8 and 2, respectively. In each docking round, we generated 9 binding modes, from which the one with the lowest binding energy (in Kcal/mol) was selected. This last step was performed 10 times for each natural ligand and compound, using different random launches. The best binding modes were chosen and LigPlot+ 2.2.4 was used to analyze the protein-ligand interactions with default parameters (Wallace et al., 1996). Visualization of the protein-ligand complexes was done with PyMOL (v. 2.4.1) (Schrödinger et al., 2020), and images were taken showing the enzymes surface and the ligands as sticks.

### Statistical analysis and interpretation of results

Absorbance and fluorescence values in the parasite growth inhibition and cytotoxicity assays were normalized to controls (Peña et al., 2015) using the following equation 1:


$$\%\;inhibition:\;100-\;100\;\times \;\frac{absorbance/fluorescence\;of\;well\;-\;positive\;control\;mean\;}{negative\;control\;mean\;-positive\;control\;mean}$$

Half maximum inhibitory (IC_50_) and half maximum toxicity concentration (TC_50_) values were determined with GraphPad Prism 7 software (version 7.00, San Diego, USA) using a nonlinear regression analysis model defined by equation 2:


$$Y\;=\;\frac{100}{\frac{1+X^{HillSlope}}{IC50^{HIllSlope}}}$$

Compounds with an IC_50_ >10× that of BNZ were considered inactive against *T. cruzi*. These values were used to calculate the selectivity index (SI) of each compound (TC_50_/IC_50_). A SI > 10 was interpreted as evidence of specific activity against *T. cruzi*, in accordance with previous works (Peña et al., 2015). Values were expressed as means with standard deviations (SD) of at least three independent experiments.

## Results

### Synthesis of 23 member 6-aminopurine-based library

The synthetic pathway designed to obtain poly-substituted derivatives of 6-aminopurine started from eight different 4,6-dialky/diarylalkylamino-5-nitropyrimidine derivatives (L2 (0-4 & 7-9)). These symmetric poly-substituted pyrimidines were then reduced to obtain the symmetric 4,6-dialky/diarylalkylamino -5-aminopyrimidine derivatives (L3 (0-4 &7-9)) using tin (II) chloride in excellent yields. At this point, we decided to carry out the cyclization step with orhtoesthers as it allows introducing structural variation in the library in a straightforward manner. Therefore, pyrimidines L3 reacted with methasulfonate and either orthoformate, trimethyl orthoformate or triphenyl orthoformate to give rise to the subfamilies a, b and c, respectively (L4 (0-4 &7-9) (a-c)) (**Supplementary material**, **Scheme 1**). To note that compound L4-7b could not be isolated from this run of reactions so the final size of the library was twenty-three rather than twenty-four as expected. These reactions were carried out at 110°C for 24 h presenting yields ranging from 10% to 82%.

### Inhibition of *T. cruzi* growth

**Figure 2:**
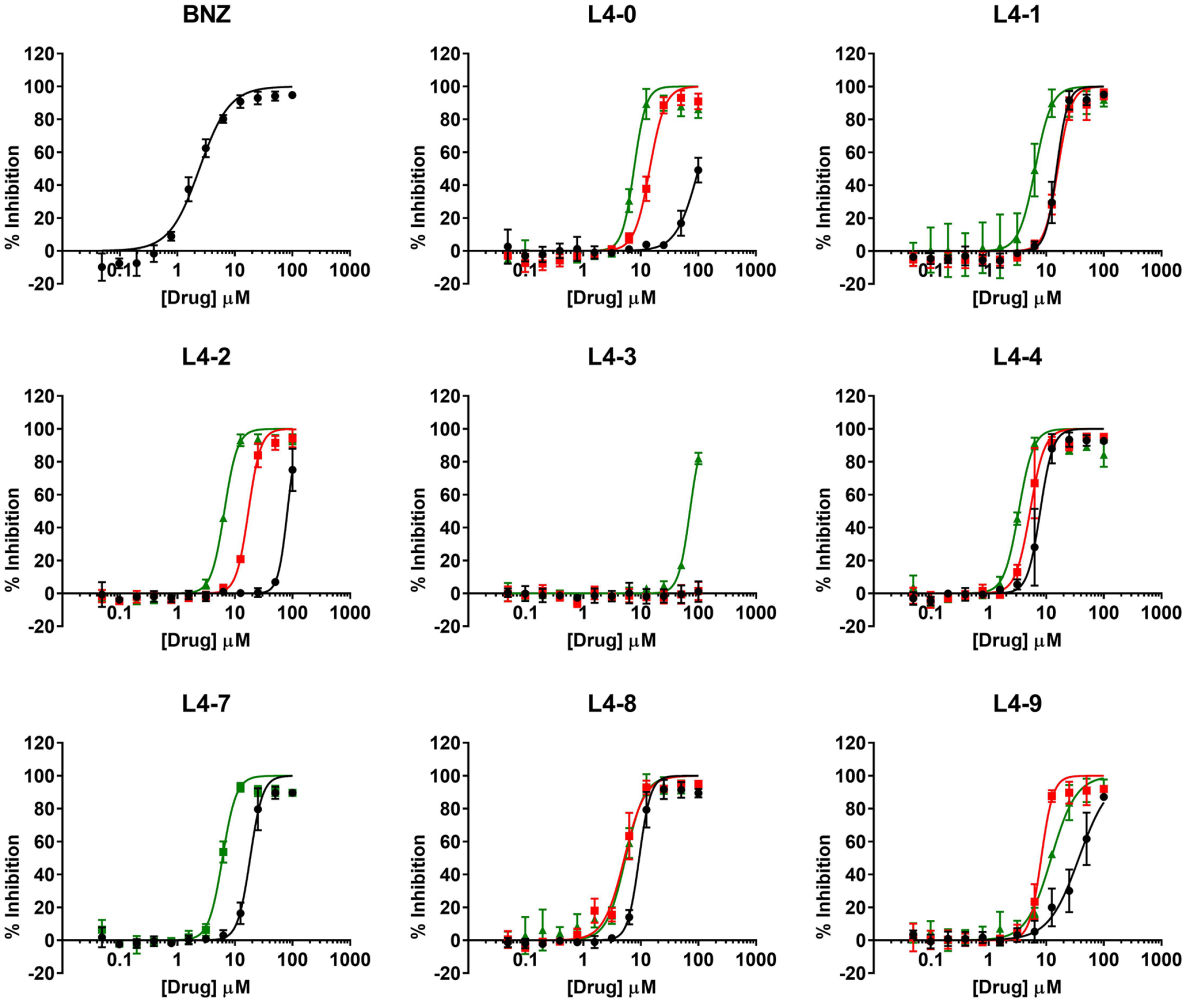
Dose-response curves of the compounds in the anti-*T. cruzi* assay. The activity curves retrieved for the three different scaffold subgroups (each identified with a letter) are shown in colors: black: a; red: b; green: c.

Compounds IC_50_ values were determined following a 1:2 dilution pattern encompassing twelve data points to construct their dose-response curves (**Figure 2**). BNZ, included as the reference drug, showed an average IC_50_ value of 2.42 ± 0.16 μM which correlated with that previously reported (Martinez-Peinado et al., 2020; Martinez-Peinado et al., 2021a; Martinez-Peinado et al., 2021b).

After evaluating the twenty-three compounds, eight of them presented an IC_50_ that was at least ten times higher than that of BNZ and were therefore considered inactive and excluded from further studies. In contrast, the other fifteen inhibited *T. cruzi* growth when compared to the reference drug BNZ **(**IC_50_ ≤10× that of BNZ; **Table 1**). The four most active compounds yielded IC_50_ values of 3.31 ± 0.17 µM (L4-4c), 5.16 ± 0.28 µM (L4-8b), 5.22 ± 0.24 µM (L4-4b), and 5.41 ± 0.32 µM (L4-8c), but none was superior to BNZ (**Table 1** and **Figure 2**).

**Table 1:** IC_50_ (μM), TC_50_ (μM), and SI values of the evaluated compounds against *T. cruzi*.

Compound	Vero cells assays			HepG2 assay	Anti-amastigote assay	
	[IC_50_] μM	[TC_50_] μM	SI	[TC_50_] μM	[IC_50_] μM	SI
**BNZ**	2.42 ± 0.16	196.90 ± 30.67	81.36	490.90 ± 267.10	1.47 ± 0.08	133.67
**L4-0a**	101.8 ± 5.84	–	–	–	–	–
L4-0b*	14.34 ± 0.58	68.16 ± 12.00	4.75	–	–	–
L4-0c*	7.64 ± 0.39	> 1,000	185.73	> 10,000	11.15 ± 0.35	127.26
L4-1a*	15.11 ± 0.56	39.26 ± 4.81	2.59	–	–	–
L4-1b*	15.89 ± 0.66	55.91 ± 5.64	3.52	–	–	–
L4-1c*	6.397 ± 0.51	> 1,000	248.09	53.96 ± 12.33	6.21 ± 0.29	255.76
**L4-2a**	81.37 ± 2.36	–	–	–	–	–
**L4-2b**	24.90 ± 0.51	–	–	–	–	–
L4-2c*	6.52 ± 0.17	> 1,000	> 1,000	> 10,000	6.19 ± 0.21	> 1,000
**L4-3a**	> 10,000	–	–	–	–	–
**L4-3b**	> 10,000	–	–	–	–	–
**L4-3c**	70.99 ± 1.29	–	–	–	–	–
L4-4a*	7.83 ± 0.37	> 1,000	139.14	> 1,000	9.36 ± 0.40	116.45
L4-4b*	5.22 ± 0.24	27.01 ± 2.00	5.17	–	–	–
L4-4c*	3.31 ± 0.17	187.50 ± 54.14	56.61	164.80 ± 38.82	4.20 ± 0.21	44.70
L4-7a*	25.17 ± 0.69	–	–	–	–	–
L4-7c*	6.06 ± 0.22	909.20 ± 564.00	150.06	550.60 ± 130.00	7.51± 0.79	121.08
L4-8a*	9.33 ± 0.35	346.30 ± 189.30	37.12	35.21 ± 5.52	–	–
L4-8b*	5.16 ± 0.28	60.11 ± 8.50	11.65	17.77 ± 0.71	–	–
L4-8c*	5.41 ± 0.32	> 1,000	600.78	> 1,000	7.61 ± 0.36	427.33
**L4-9a**	36.84 ± 2.38	–	–	–	–	–
L4-9b*	8.16 ± 0.35	> 1,000	137.56	372.20 ± 51.70	8.87 ± 0.42	126.59
L4-9c*	12.19 ± 0.67	> 10,000	> 10,000	>10,000	28.70 ± 2.34	–

^*^Compounds with an IC_50_ ≤ 10x that of BNZ.

### Identification of compounds with specific activity against the parasite

Those fifteen compounds that inhibited *T. cruzi* growth were furtherly assessed to determine whether such inhibition was specific against the parasite by means of a Vero cell toxicity assay. The recorded TC_50_ value of BNZ in this assay was 196.90 ± 30.67 µM (**Table 1** and **Figure S1**), which matches previously reported results (Martinez-Peinado et al., 2020).

Eleven compounds (L4-0c, L4-1c, L4-2c, L4-4a, L4-4c, L4-7c, L4-8a, L4-8b, L4-8c, L4-9b and L4-9c) had SI values > 10. However, only eight had SI values higher than that of BNZ (SI = 81.36) (**Table 1**).

### HepG2 cell toxicity assay

Toxicity to HepG2 of the eleven compounds that were specifically active against the parasite was then evaluated (**Figure S2**) (Cho et al., 2015; Martinez-Peinado et al., 2021b). TC_50_ values of BNZ and DTX in this cell line were respectively 490.90 ± 267.10 µM and 1.29 ± 0.40 μM (**Table 1**). While five compounds L4-1c, L4-4c, L4-8a, L4-8b and L4-9b presented a TC_50_ lower than BNZ, only compounds L4-8a (TC_50_ = 35.21 ± 5.52 μM) and L4-8b (TC_50_ = 17.77 ± 0.71 μM) were found to be more toxic than the established 50 μM threshold (Martinez-Peinado et al., 2021b) and thus were excluded from being further evaluated (**Table 1**).

### Inhibition of amastigotes growth

**Figure 3:**
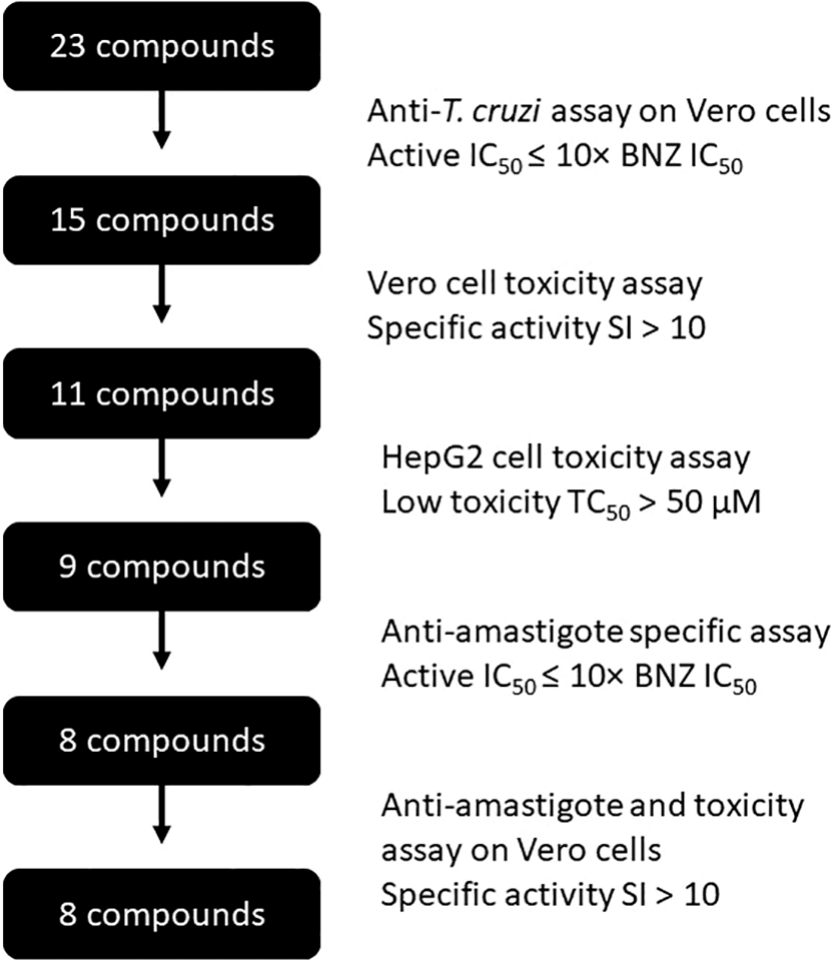
Flow-chart describing the compounds progression steps.

**Figure 4:**
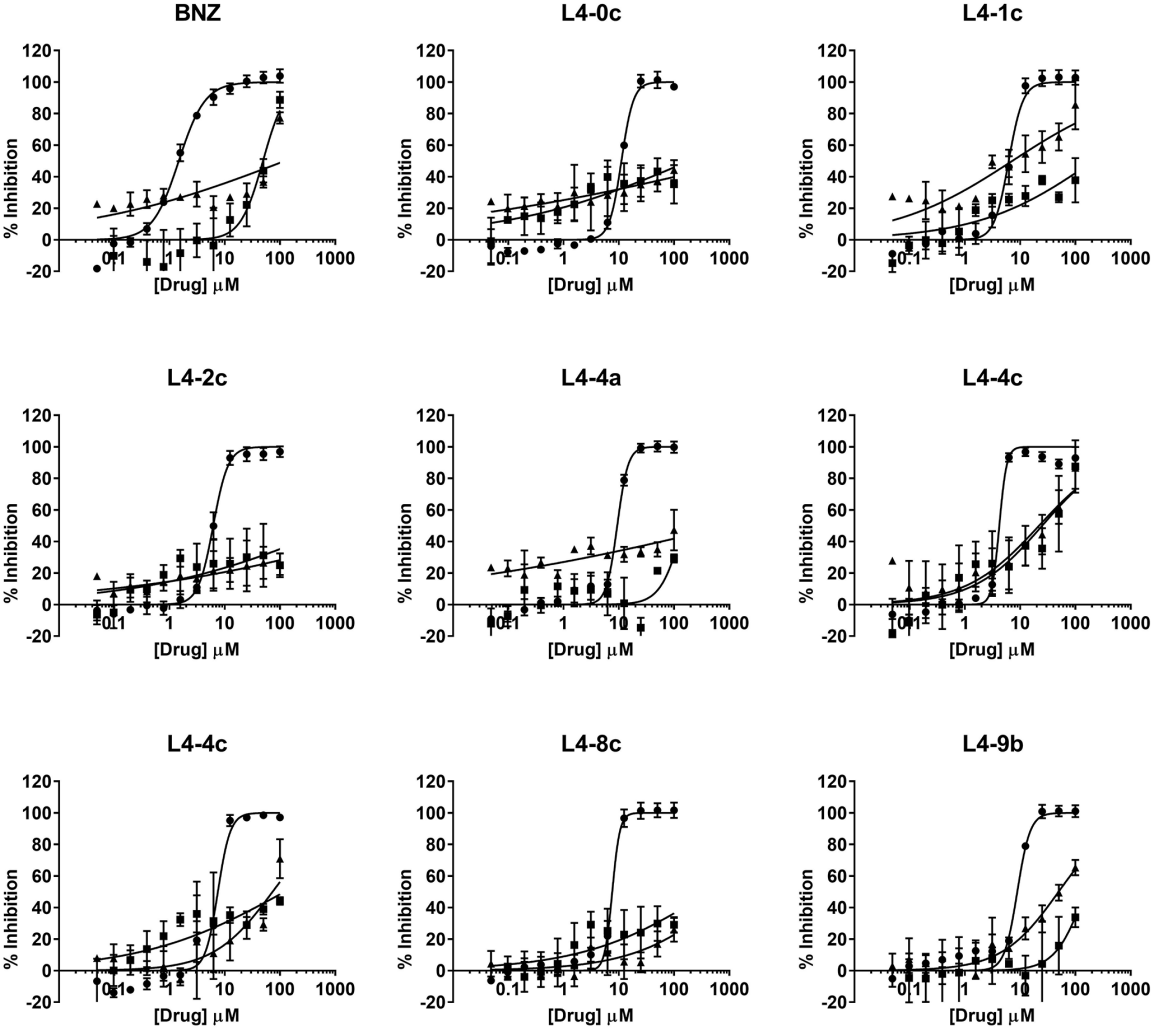
Dose-response curves of selected compounds based on their anti-parasitic activity. Anti-amastigote activity is represented by circles, Vero cell toxicity by squares, and HepG2 cells toxicity by triangles.

The nine anti-*T. cruzi* compounds found to be non-toxic to HepG2 cells were tested to determine their specific activity against amastigotes (**Figure 3**), the intracellular replicative form of *T. cruzi* in mammals and a major target for any prospective drug against chronic Chagas disease. The IC_50_ of BNZ in these assays was 1.47 ± 0.08 μM (**Table 1**). Out of the nine compounds studied, eight were found to be active against amastigotes (IC_50_ ≤ 10× that of BNZ): L4-0c, L4-1c, L4-2c, L4-4a, L4-4c, L4-7c, L4-8c and L4-9b. On the other hand, compound L4-9c was inactive. None of the compounds was discarded due to unspecific activity against amastigotes (**Table 1**), and none was found to be more active than BNZ (**Figure 4**). Remarkably, when comparing Vero cells TC_50_ toxicities and anti-amastigote IC_50_ values, L4-1c, L4-2c and L4-8c showed higher SI windows than BNZ (**Table 1**).

### Computational analysis

The final eight compounds with specific anti-amastigote activity were studied *in silico* to identify their probable targets in the purine salvage pathway. For this, we compared the free binding energies and docking positions between compounds and the enzymes natural ligands, as these would compete for the active binding site. All compounds showed lower binding energies than the natural ligands when docked to *Tc*HGPRT, *Tc*APRT, *Tc*AK, *Tc*IGNH and *Tc*MTAP (**Table S1**). Among them, L4-8c exhibited the lowest energy values when docked to *Tc*IGNH (ΔG = -10.8 Kcal/mol) and *Tc*MTAP (ΔG = -10.7 Kcal/mol) (**Table S1**). Compounds L4-0c, L4-1c, L4-4a and L4-8c exhibited the lowest energy value when docked against *Tc*IGNH, whereas compounds L4-2c, L4-4c and L4-7c did it against *Tc*MTAP enzyme. Compound L4-9b showed the lowest binding energy values against both *Tc*IGNH and *Tc*MTAP. However, the binding energy values of the natural ligands of both enzymes were similar to that of the compounds, which led to low energy differences between both. While most compounds showed the capacity to interact with several enzymes of the pathway, the highest energy differences were shown with *Tc*APRT (**Table 2**), except for compound L4-9b for which it was with *Tc*HGPRT (**Table 2**), suggesting that these two enzymes are preferentially inhibited.

**Table 2:** Differences between the free binding energies of natural ligands and “hit” compounds with enzymes from the purine salvage pathway of *T. cruzi*.

Enzyme	Natural Ligand	Energy Differences with the Compounds (Kcal/mol)							
		L4-0c	L4-1c	L4-2c	L4-4a	L4-4c	L4-7c	L4-8c	L4-9b
***Tc*HGPRT**	Hypoxanthine	-3.1	-3.0	-3.0	-3.1	-3.1	-2.7	-3.6	-3.1
	Guanine	-2.6	-2.5	-2.5	-2.6	-2.6	-2.2	-3.1	-2.6
***Tc*APRT**	Adenine	-3.1	-3.2	-3.5	-3.1	-3.5	-3.6	-4.0	-2.5
***Tc*AK**	Adenosine	-1.6	-1.6	-1.4	-0.8	-1.4	-1.6	-1.8	-1.5
***Tc*IGNH**	Inosine	-1.6	-1.6	-0.6	-1.5	-1.0	-1.3	-2.5	-1.6
	Guanosine	-1.2	-1.2	-0.2	-1.1	-0.6	-0.9	-2.1	-1.2
***Tc*IMPDH**	IMP	2.7	3.8	3.6	2.0	3.2	3.8	2.9	3.5
***Tc*IAGNH**	Inosine	2.3	1.9	2.1	2.2	2.0	1.8	1.7	2.9
	Adenosine	2.3	1.9	2.1	2.2	2.0	1.8	1.7	2.9
	Guanosine	2.4	2.0	2.2	2.3	2.1	1.99	1.8	3.0
***Tc*ADSL**	Adenylosuccinate	0.7	2.0	0.6	1.1	1.0	1.7	0.3	1.0
	SAICAR	0.5	1.8	0.4	0.9	0.8	1.5	0.1	0.8
***Tc*MTAP**	Methylthioadeno-sine	-1.1	-2.7	-2.5	-1.7	-2.7	-2.8	-3.1	-2.3

**Figure 5:**
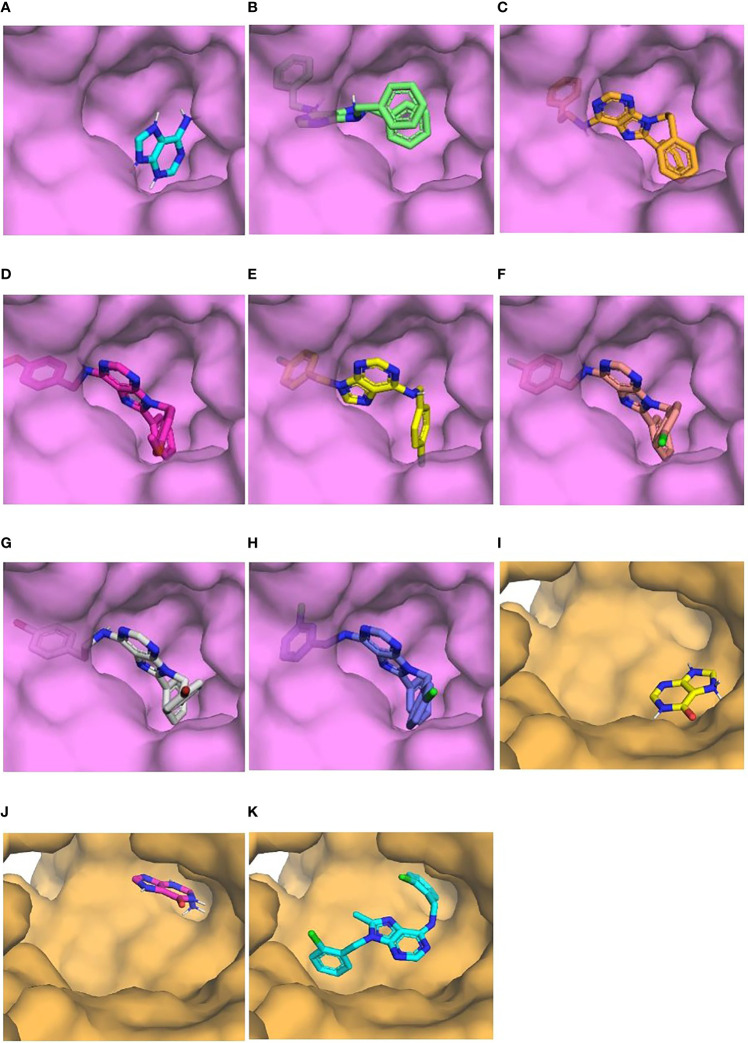
Binding of natural ligand and “hits” with the enzymes on which they had the highest energy differences. Surface view of the binding pocket of *Tc*APRT (purple) and *Tc*HGPRT (orange). Adenine **(A)**, L4-0c **(B)**, L4-1c **(C)**, L4-2c **(D)**, L4-4a **(E)**, L4-4c **(F)**, L4-7c **(G)**, L4-8c **(H)**, Hypoxanthine **(I)**, Guanine **(J)**, L4-9b **(K)**.

**Figure 6:**
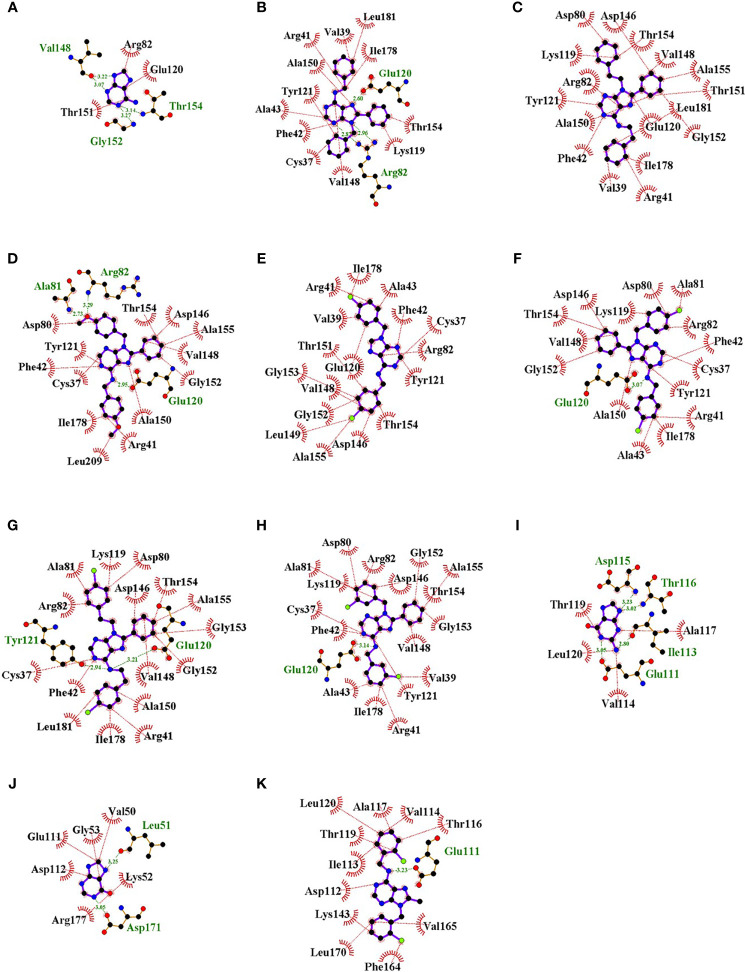
Interactions of the active site residues of the enzymes *Tc*APRT and *Tc*HGPRT with the natural ligands and “hit” compounds. Representative hydrophobic interactions and hydrogen bonds are represented in red and green dot lines, respectively. Adenine **(A)**, L4-0c **(B)**, L4-1c **(C)**, L4-2c **(D)**, L4-4a **(E)**, L4-4c **(F)**, L4-7c **(G)**, L4-8c **(H)**, Hypoxanthine **(I)**, Guanine **(J)**, L4-9b **(K)**. Ligands A-H are interacting with *Tc*APRT and I-K with *Tc*HGPRT.

Visualization of the interactions of compounds and those two enzymes showed that compounds bound to the active site occupying a larger fraction of the cavity than natural ligands (**Figure 5**). Interestingly, substitutions at C8 and N9 of the purine ring of all compounds except L4-0c filled the location of adenine in *Tc*APRT (**Figure 5**). Similarly, the substitution at C6 of compound L4-9b was found in the binding site of hypoxanthine or guanine in *Tc*HGPRT (**Figure 5**). In general, compounds showed more hydrophobic interactions and less hydrogen bonds than the natural ligands. In the *Tc*APRT model, its natural ligand (adenine) formed three hydrophobic interactions with arginine R82, glutamic acid E120 and threonine T151, and four hydrogen bonds with valine V148, glycine G152 and T154 (**Figure 6A**). Residues R82 and E120 formed hydrophobic interactions, whereas in L4-0c and L4-2c these residues were involved in hydrogen bonds (**Figures 6A, B, D**). These two amino acids were also found in the interactions with L4-1c, L4-4a, L4-4c,L4-7c and L4-8c (**Figures 6C, E–H**). Compounds L4-1c and L4-4a did not present hydrogen bonds (**Figures 6C, E**). In the *Tc*HGPRT model, hypoxanthine and guanine did not interact with the same residues since they were located at different sites of the enzyme cavity and only shared the interaction with E111 (**Figures 6I, J**). Compound L4-9b shared several interactions with hypoxanthine and guanine, such as those observed with residues E111, aspartic acid D112, isoleucine I113, and T116 of the enzyme (**Figure 6K**).

## Discussion

The purine metabolism holds striking differences between *T. cruzi* and its mammalian hosts. Therefore, it has been considered a niche of potential therapeutic targets. Several studies have reported anti-*T. cruzi* activity of purine analogs in *in vitro* and *in vivo* models. Some of those compounds decreased parasitemia and reduced mortality but were unable to induce sterile cure in mouse models (Hulpia et al., 2018, Cardoso-Santos et al., 2021). Thus, new purine chemotypes would be very welcome to progress in the development of new therapeutic options for Chagas disease.

The collection evaluated in this paper is partly based on that reported by Martinez-Peinado (Martinez-Peinado et al., 2021b) but with 6 aminopurine as the core structure of the library. We hypothesized that the nitrogen in position 6 could improve the molecular interaction of these compounds with their target enzyme as it can act as both hydrogen bond donor and acceptor while the oxygen at that position in the previous library can act just as hydrogen bond acceptor. Previously, two out of eighty-one compounds (2.47%) were identified as potential drug candidates (Lorente et al, 2021; Martinez-Peinado et al., 2021b). In the current study, eight out of twenty-three (34.78%) molecules demonstrated specific activity against *T. cruzi* amastigotes (**Figure 3**), indicating that the amino group at position 6 favored the anti-parasitic activity of the purine analogs *in vitro*, possibly by improving diffusion in the cytoplasm and extracellular medium as well as facilitating the formation of water-mediated hydrogen bonds between compounds and their molecular targets.

Interestingly, all compounds from the L4-3 family, characterized by the absence of benzene rings in positions C6 and N9, showed no activity against the parasite (**Figure 1**). Similarly, all compounds belonging to group c, characterized by the presence of a benzyl group in C8 of the purine ring, showed low toxicity on Vero cells and were active against the parasite. Taken together, these findings suggest that the presence of benzene rings in positions C6 and N9, and a benzyl group at C8, are important for the selective interaction of inhibitors with *T. cruzi* enzymes in the purine salvage pathway, but not with its mammal counterparts, thus leading to specific anti-parasitic activity. Aromatic rings are known to increase the activity of purine analogues against malaria parasites (Harmse et al., 2001), and *Trypanosoma brucei* (Pineda de las Infantas et al., 2015). The presence of benzyl groups in position C6 of the purine ring has also been previously identified in active analogs against *T. cruzi* (Martinez-Peinado et al., 2021b).

Among the eight compounds selected, five carried the benzene ring group at position C8 illustrating the relevance of this substituent. Family by family, L4-0c was the only selected from L4-0. Characterized by the presence of three benzene rings in positions C6, C8 and N9, it had some of the poorer anti-amastigote IC_50_ retrieved (11.15 ± 0.35 μM), as well as out of fitting range TC_50_ values on Vero and HepG2 cells (**Table 1**). Surprisingly, microscopy-based observation of the compound in assay medium revealed that it failed to be completely dissolved (**Figure S3**). Similarly, the presence of crystals was observed in assay medium-dissolved compounds L4-8c and L4-9b, likely affecting the reliability of the activity results retrieved with all three of them.

Compared to L4-0c, L4-1c substitutions in C6 and N9 contain an extra carbon atom that confers a larger chemical structure, which may benefit its solubility. Compared to L4-1c, L4-1a and L4-1b showed poorer anti-*T. cruzi* activity and higher toxicity to Vero cells highlighting the importance of the extra C8-benzyl (**Table 1**). Regarding family L4-2, only compound L4-2c, again in the scaffold group c, displayed specific anti-*T. cruzi* activity. In comparison with L4-1c, L4-2c has two *p*-methoxyphenyl radicals in positions C6 and N9 (**Figure 1**).

Two components of family L4-4, L4-4a and L4-4c showed specific anti-amastigote activity. Both have *p*-chlorobenzyl substituents at positions C6 and N9, but again it was L4-4c with a benzene ring in position C8 which yielded better results (**Figure 1**). In fact, it was the most active against *T. cruzi*, performing very close to BNZ in the primary antiparasitic assay of the cascade (**Table 1**). In contrast, L4-4b presented a similar anti-*T. cruzi* IC_50_ to that of L4-4a and L4-4c, but the presence of a methyl group at C8 seemed to increase toxicity on Vero cells and it was discarded for not complying with the SI threshold.

L4-7c has a similar structure to that of L4-1c, but with benzyl *p*-bromide groups instead of a benzyl *p*-chloride substitution at C6 and N9 positions. Both compounds showed similar IC_50_ values (**Table 1**).

Our *in silico* results suggest that *Tc*APRT and *Tc*HGPRT are the two most likely targets of the studied compounds. Visualization of docking results revealed that compounds tend to cover a larger room of the active site than the natural ligands (**Figure 5**). For instance, in *Tc*APRT model, compounds aromatic groups in positions C8 and N9, linked to specific anti-parasitic effects, seem to occupy a similar position in the active site of *Tc*APRT as adenine aromatic groups. The larger purine analogs tested here could be filling the site for natural ligands adenine and phosphate, preventing their entry to the active site and their catalysis to form AMP. Compounds interactions with *Tc*APRT were characterized by the presence of hydrophobic interactions with representative residues such as T154 and R82. Our hypothesis about the nitrogen in position 6 that can act as both hydrogen bond donor and acceptor can be clearly seen with compound L4-7c, one of the most potent compounds of the library that would form two hydrogen bonds between the exocyclic nitrogen at position 6 and residues Y121 and E120 of the enzyme *Tc*APRT according to LigPlot predictions (**Figure 6**). That means that the amino group act as hydrogen bonding donor and acceptor. Moreover, exocyclic amino groups at position 6 of active inhibitors as L4-2c, L4-4c and L4-8c act as hydrogen bonding donor in their interaction with E120 of *Tc*APRT (**Figure 6**).

Adenine metabolism plays a key role in several cellular processes, including energy production, nucleic acid metabolism and synthesis of NAD^+^ and coenzyme A, and stress responses (García-Huertas et al., 2017). An imbalance in adenine can lead to alterations in energy metabolism that play an important role in mediating stress tolerance. This indicates a connection between purine, ROS, and stress responses, all of them identified as suitable molecular targets in *T. cruzi* (Beltran-Hortelano et al., 2022). The inhibition of this particular reaction is therefore likely to indirectly disrupt other essential metabolic pathways, apart from nucleic acid synthesis. Interestingly, Garcia-Huertas and co-authors found that APRT enzyme was down-regulated in BNZ resistant *T. cruzi* parasites (García-Huertas et al., 2017). Thus, the activity of these compounds on BNZ resistant strains should be further studied.

Regarding *Tc*HGPRT, the docking results suggest that L4-9b would bind in a similar fashion as active analogs formerly described (Martinez-Peinado et al., 2021b). Other work identified purine analogs with electronegative atoms such as chlorine that also produced similar bindings (Eakin et al., 1997). However, experimental output of L4-9b might not be totally reliable due to the observation of crystals when dissolved in assay medium. Anyhow, according to the docking analysis, all the prioritized compounds would be acting as true inhibitors instead of alternative substrates as they presented substitutions at N9 which would not allow the incorporation of phosphoribosyl pyrophosphate to the purine base in both APRT and HGPRT enzymes.

Models indicate that compounds generally established more hydrophobic interactions than hydrogen bonds with *Tc*APRT and *Tc*HGPRT. However, this is likely influenced by the non-inclusion of water molecules in our docking analysis. Since the "hit" compounds lack polar hydrogens but have a large number of heteroatoms, they may form water-mediated hydrogen bonds with other amino acids.

Collectively, our results confirm that inhibitors of the purine salvage pathway are promising candidates for the development of alternative treatments for Chagas disease. Before moving to *in vivo* experiments, it will be very important to address their activity against other genetically diverse strains of the parasite as well as against BNZ-resistant isolates. Additionally, experimental confirmation that the compounds are acting as specific inhibitors of these two enzymes should also be pursued.

## Data availability statement

The original contributions presented in the study are included in the article/**Supplementary Material**. Further inquiries can be directed to the corresponding authors.

## Author contributions

Conceptualization: JA-P, NM-P. Data curation: BB, JG-F, NM-P. Data analysis: BB, JG-F, NM-P. Supervision of activities: JA-P, NM-P, MJPdlIyV, IM. Methodology: BB, JG-F, NM-P, JA-P. Chemical Library synthesis: LG, MJPdlIyV, JD-M. Laboratory investigation: BB, JG-F, NM-P. Software: BB, AR-L. Resources: JA-P, JG. Wrote the draft manuscript: BB, JG-F, AR-L, NM-P, JA-P, MJPdlIyV, JD-M. Review and editing of manuscript: BB, JG-F, AR-L, NM-P, JA-P, LG, MJPdlIyV, JD-M. All authors read and approved the final manuscript.

## Supplementary material

Scheme 1 Synthetic pathway to obtain the twenty three member library based on 6-aminopurine.
