# Supplementary material for: Identification of compounds with activity against Trypanosoma cruzi within a collection of synthetic nucleoside analogs
Source: Front Cell Infect Microbiol. 2023 Jan 13;12:1067461. doi: 10.3389/fcimb.2022.1067461 (PMC9880260; doi:10.3389/fcimb.2022.1067461)
Supplement: Scheme 1 — Synthetic pathway to obtain the twenty three member library based on 6-aminopurine. [file DataSheet_1.docx]

Supplementary Material

Identification of compounds with activity against *Trypanosoma cruzi* within a collection of synthetic nucleoside analogs

Berta Barnadas^1^, Nieves Martínez-Peinado^1,2^, Laura Córdoba Gómez^3^, Albert Ros-Lucas^1,4^, Juan Carlos Gabaldón-Figueira^1^, Juan J. Diaz-Mochon^3,5,6^, Joaquim Gascón^1,4^, Ignacio J. Molina^7^, María José Pineda de las Infantas^3,*^, Julio Alonso-Padilla^1,4*^

*** Correspondence:** Julio Alonso Padilla: julio.a.padilla@isglobal.org ; María José Pineda de las Infantas mjpineda@ugr.es

**Figure S1**. **Dose-response curves of the compounds in the Vero cell toxicity assay**. Activity curves of the three scaffold subgroups are shown in colors: black, a; red, b; green, c.


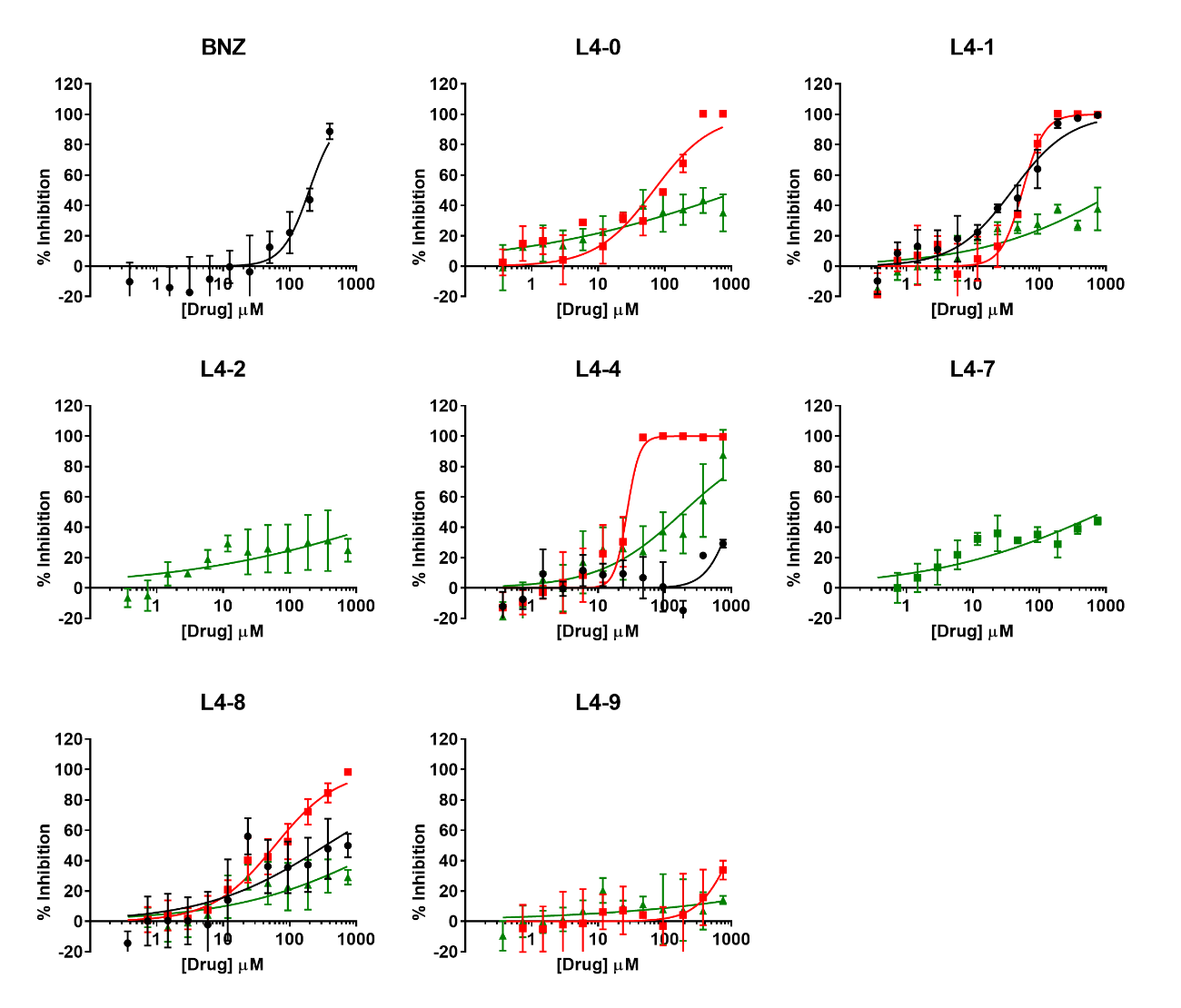


**Figure S2**. **Dose-response curves of the compounds in the HepG2 cell toxicity assay.** The different scaffold subgroups are shown in colors: black, a; red, b; green, c.

**
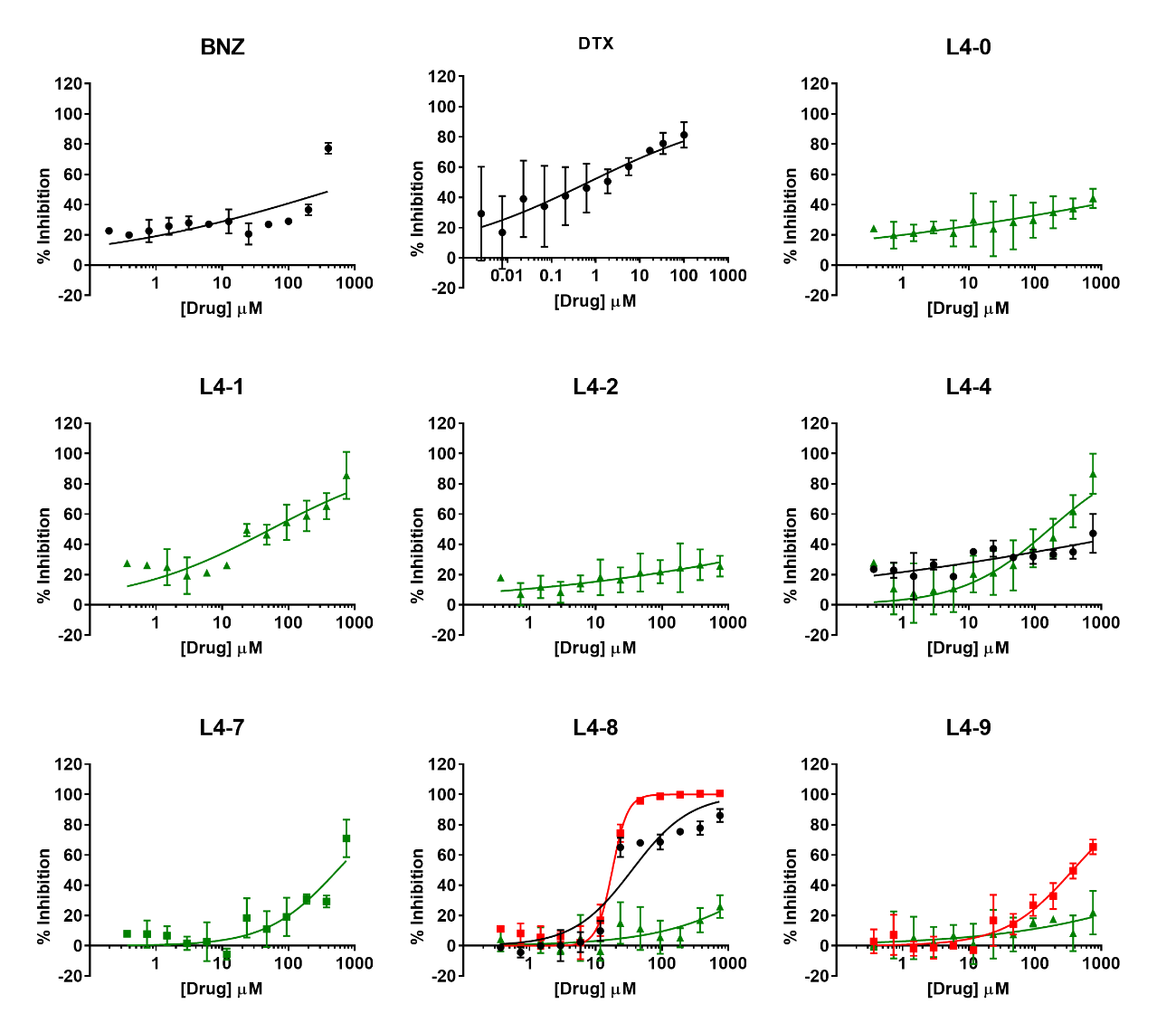
**

**Figure S3**. **Crystals of the compounds [750 μM] formed during the assays.** The eight compounds selected through the discovery cascade are shown in bold. Besides, those indicated with an asterisk too had solubility issues. BNZ is shown at 400 μM.

| 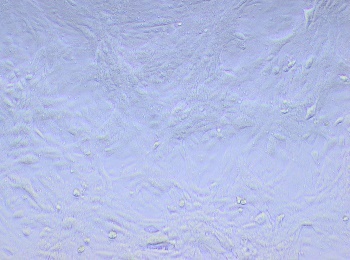Control | 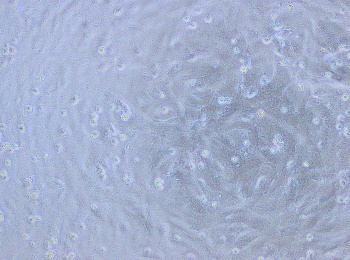BNZ | **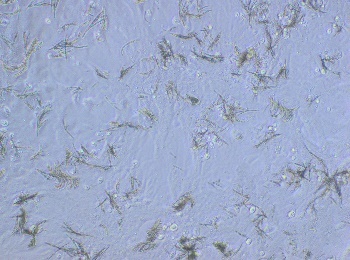L4-0a*** | 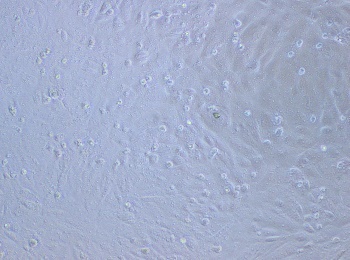L4-0b | 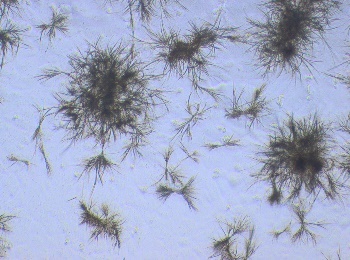L4-0c |
| --- | --- | --- | --- | --- |
| 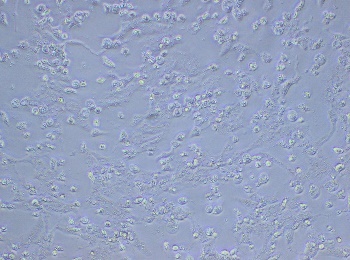L4-1a | 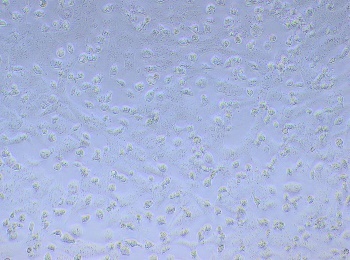L4-1b | **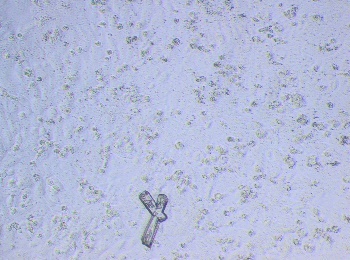L4-1c** | 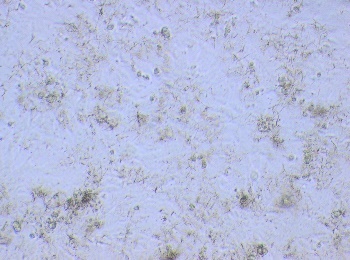L4-2a | 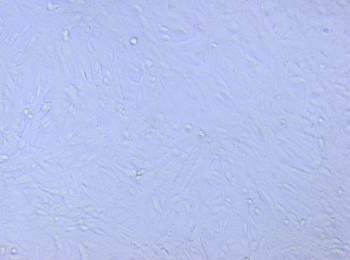L4-2b |
| **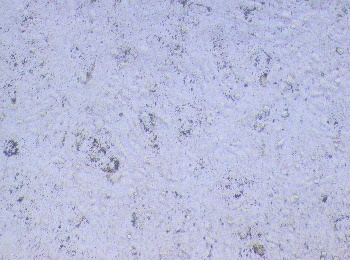L4-2c** | 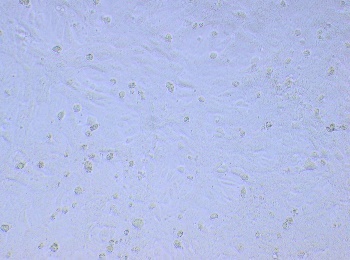L4-3a | 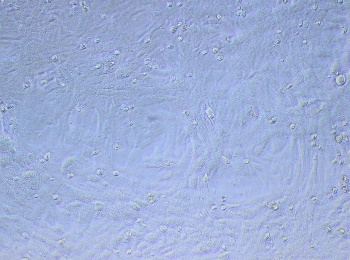L4-3b | 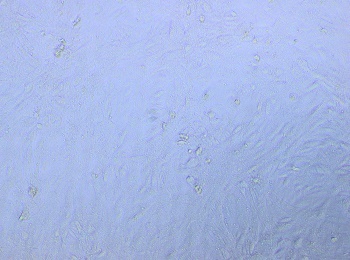L4-3c | **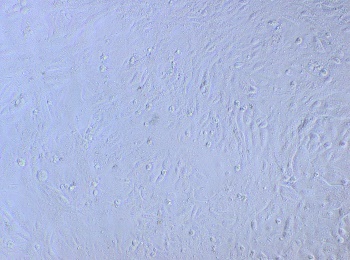L4-4a** |
| 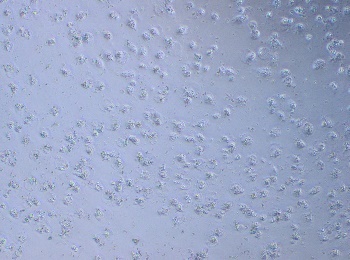L4-4b | **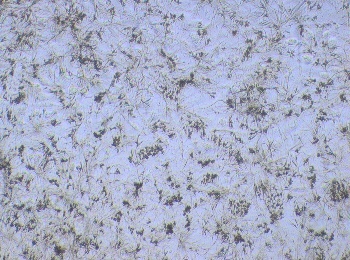L4-4c** | 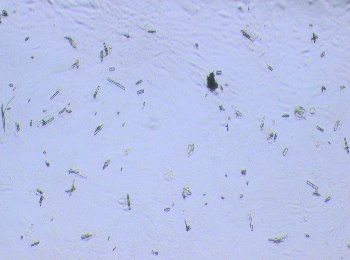L4-7a | **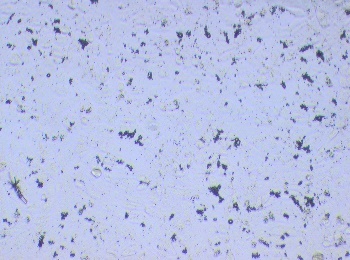L4-7c** | 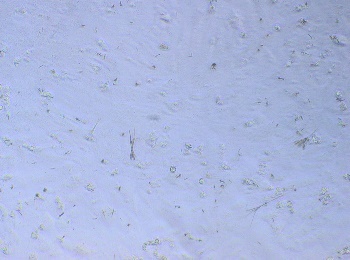L4-8a |
| 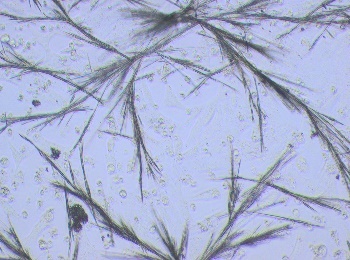L4-8b | **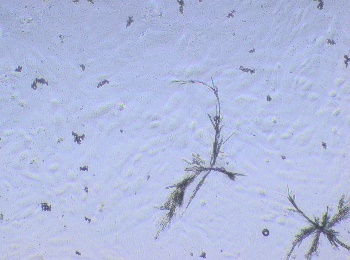L4-8c*** | 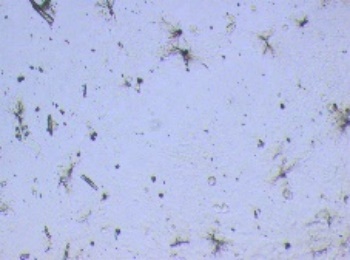L4-9a | **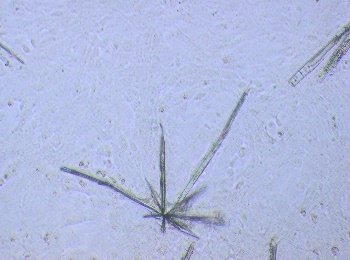L4-9b*** | 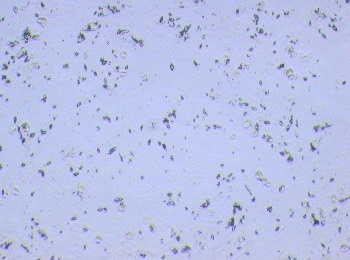L4-9c |

**Table S1. Free binding energies of molecular docking between natural ligands and “hit” compounds with enzymes from the purine salvage pathway of *T. cruzi*.**

| **Enzyme** | **Natural Ligand** | **Energy Binding with Natural Ligand (Kcal/mol)** | **Energy Binding with the Compounds (Kcal/mol)** | | | | | | | |
| --- | --- | --- | --- | --- | --- | --- | --- | --- | --- | --- |
|  |  |  | **L4-0c** | **L4-1c** | **L4-2c** | **L4-4a** | **L4-4c** | **L4-7c** | **L4-8c** | **L4-9b** |
| ***Tc*HGPRT** | Hypoxanthine | -5.0 | -8.1 | -8.0 | -8.0 | -8.1 | -8.1 | -7.7 | -8.6 | -8.1 |
|  | Guanine | -5.5 |  |  |  |  |  |  |  |  |
| ***Tc*APRT** | Adenine | -5.8 | -8.9 | -9.0 | -9.3 | -8.9 | -9.3 | -9.4 | -9.8 | -8.3 |
| ***Tc*AK** | Adenosine | -8.1 | -9.7 | -9.4 | -9.5 | -8.9 | -9.5 | -9.7 | -9.9 | -9.6 |
| ***Tc*IGNH** | Inosine | -8.3 | -9.9 | -9.9 | -8.9 | -9.8 | -9.3 | -9.6 | -10.8 | -9.9 |
|  | Guanosine | -8.7 |  |  |  |  |  |  |  |  |
| ***Tc*IMPDH** | IMP | -10.3 | -7.6 | -6.5 | -6.7 | -8.3 | -7.1 | -6.5 | -7.4 | -6.8 |
| ***Tc*IAGNH** | Inosine | -9.6 | -7.3 | -7.7 | -7.5 | -7.4 | -7.6 | -7.8 | -7.9 | -6.7 |
|  | Adenosine | -9.6 |  |  |  |  |  |  |  |  |
|  | Guanosine | -9.7 |  |  |  |  |  |  |  |  |
| ***Tc*ADSL** | Adenylosuccinate | -8.0 | -7.3 | -6.0 | -7.4 | -6.9 | -7.0 | -6.3 | -7.7 | -7.0 |
|  | SAICAR | -7.8 |  |  |  |  |  |  |  |  |
| ***Tc*MTAP** | Methylthioadenosine | -7.6 | -8.7 | -10.3 | -10.1 | -9.3 | -10.3 | -10.4 | -10.7 | -9.9 |

1. **Synthesis and characterization of the chemical collection**
   1. **General**

Reaction courses and products mixtures where routinely monitored by TLC on silica gel Merck 60-200 mesh silica gel. Melting points were determined on a Stuart Scientific SMP3 apparatus and are incorrected. ^1^H-NMR spectra were obtained in CDCl_3_, solution on a Varian Direct Drive (400 MHz and 500 MHz). Chemical shifts (δ) are given in ppm upfield from tetramethylsilane. ^13^C-NMR spectra were obtained in CDCl_3_, on a Varian Direct Drive (125 MHz). All products reported showed ^1^H-NMR and ^13^C-NMR spectra in agreement with the assigned structures. Mass spectra were obtained by electrospray (ESY) with a LCT Premier XE Micromass Instrument (High resolution mass spectrometry).

- **1.2. General procedure for the preparation of compounds L3(0-9):**

A solution of **L2(0-9)** (5 mmol, 1 equiv.) in EtOH (10 ml) containing SnCl_2_.2H_2_O (25 mmol, 5 equiv.), was refluxed for 1 h, monitoring the reaction by TLC. The mixture was then cooled at room temperature and NaHCO_3_ was added until pH 8 was reached. After two extractions with EtOAc (10 ml for each one), the organic phase was washed with an aq. saturated solution of NaCl (2x25 mL) and dried on Na_2_SO_4_. Evaporation under vacuum gave a solid that was purified by column chromatography eluting with ethyl acetate/petroleum ether solutions.

- **1.3. General procedure for the preparation of compounds L4(0-9) (1-c):**

A solution of **L3(0-9)** (1 equiv.), with un excess (500 µL) of the corresponding trialkylorthoesthers and methanesulfonic acid (0.2 equivalent) was heated to 110° C for 24 h, monitoring the reaction by TLC. The mixture was then cooled at room temperature. After two extractions with CH_2_Cl_2_ the organic phase was washed with an aq. saturated solution of NaCl and dried on Na_2_SO_4_. Evaporation under vacuum gave a solid that was purified by column chromatography eluting with ethyl acetate/petroleum ether solutions.

- **1.4. Characterization of L4(0-9) (a-c) compounds:**

N,9-dibenzyl-9H-purin-6-amine (L4a): White solid, yield 57%, bp 175˚C. δH (400.45 MHz, CDCl_3_): 8.45 (1H, s, NC*H*N)*,* 7.61 (1H, s, NC*H*N)*,*7.38-7.27 (10H, m, Ph x2), 6.41 (1H, bs, N*H*), 5.35 (2H, s, NHC*H_2_*Ph), 4.88 (2H, s, NC*H_2_*Ph). δC (100.70 MHz, CDCl_3_): 154.64, 153.50, 149.80, 139.97, 138.47, 135.66, 129.19, 128.81, 128.55, 127.93, 127.87, 127.64, 119.65, 47.34, 44.68. ES+HRMS: Calculated M+H=316.1562 C_19_H_18_N_5_. Obtained: 316.1576.

N,9-dibenzyl-8-methyl-9H-purin-6-amine (L4b): Yellow solid, yield 42%. δH (400.45 MHz, CDCl_3_): 8.41 (1H, s, NCHN), 7.41-7.15 (10H, m, Ph x2), 5.36 (2H, s, NHCH_2_Ph), 4.91 (2H, s, NCH_2_Ph), 2.45 (3H, s, CCH_3_). δC (100.70 MHz, CDCl_3_): 153.24, 149.48, 138.37, 135.70, 132.61, 131.01, 129.14, 128.81, 128.25, 127.97, 127.65, 127.17, 118.28, 46.07, 38.88, 14.38. ES+HRMS: Calculated M+H=330.1719 C_20_H_20_N_5_. Obtained: 330.1717.

N,9-dibenzyl-8-phenyl-9H-purin-6-amine (L4c): White solid, yield 49%, bp 162˚C. δH (400.45 MHz, CDCl_3_): 8.46 (1H, s, NCHN), 7.59-7.06 (15H, m, Ph x3), 6.24 (1H, bs, NH), 5.48 (2H, s, NHCH_2_Ph), 4.92 (2H, s, NCH_2_Ph). δC (100.70 MHz, CDCl_3_): 151.33, 138.39, 136.51, 133.03, 130.38, 129.71, 129.23, 129.03, 129.00, 128.85, 128.49, 128.06, 127.99, 127.72, 126.81, 119.45, 49.84, 47.15. ES+HRMS: Calculated M+H=392.1875 C_25_H_22_N_5_. Obtained: 392.1881.

N,9-diphenethyl-9H-purin-6-amine **(L4-1a):** Pink solid, yield 85 %, bp 182^o^ C. δH (400.45 MHz, CDCl_3_): 8.44 (1H, s, NC*H*N)*,* 7.34 (1H, s, NC*H*N)*,* 7.32-7.07 (10H, m, Ph x2), 6.00 (1H, bs, N*H*), 4.45 (2H, t, J=5.60, NC*H_2_*CH_2_Ph), 3.98 (2H, m, NHC*H_2_*CH_2_Ph), 3.19 (2H, t, J=5.60, NCH_2_C*H_2_*Ph), 3.03 (2H, t, J=5.60, NHCH_2_C*H_2_*Ph). δC (100.70 MHz, CDCl_3_): 153.30, 153.04, 140.07, 138.95, 137.52, 128.99, 128.96, 128.85, 128.75, 127.19, 126.65, 119.77, 43.58, 36.33, 36.08. ES+HRMS: Calculated M+H=344.1875 C_21_H_22_N_5_. Obtained: 344.1879.

8-methyl-N,9-diphenethyl-9H-purin-6-amine **(L4-1b):** Yellow solid, yield 75 %. δH (400.45 MHz, CDCl_3_): 8.41 (1H, s, NC*H*N)*,* 7.33-7.00 (10H, m, Ph x2), 4.35 (2H, t, J=6.80, NC*H_2_*CH_2_Ph), 3.95 (2H, m, NHC*H_2_*CH_2_Ph), 3.13 (2H, t, J=6.80, NCH_2_C*H_2_*Ph), 3.04 (2H, t, J=6.80, NHCH_2_C*H_2_*Ph), 2.09 (3H, s, C*H_3_*). δC (100.70 MHz, CDCl_3_): 153.60, 152.63, 148.95, 143.11 139.32, 137.67, 129.00, 128.97, 128.94, 128.54, 127.20, 126.38, 117.60, 44.82, 42.40, 35.92, 21.37. ES+HRMS: Calculated M+H=358.2032 C_22_H_24_N_5_. Obtained: 358.2060.

N,9-diphenethyl-8-phenyl-9H-purin-6-amine **(L4-1c):** Yellow solid, yield 76 %. δH (500 MHz, CDCl_3_): 8.46 (1H, s, NC*H*N)*,* 7.50-6.91 (15H, m, Ph x3), 5.99 (1H, bs, N*H*), 4.49 (2H, t, J=7.50, NC*H_2_*CH_2_Ph), 3.97 (2H, m, NHC*H_2_*CH_2_Ph), 3.10 (2H, t, J=7.50, NCH_2_C*H_2_*Ph), 3.02 (2H, t, J=7.50, NHCH_2_C*H_2_*Ph). δC (100.70 MHz, CDCl_3_): 154.40, 152.97, 150.10, 139.00, 137.47, 130.05, 129.11, 128.98, 128.89, 128.84, 128.73, 128.71, 126.94, 126.59, 119.53, 45.33, 42.19, 36.13, 35.79. ES+HRMS: Calculated M+H=420.2188 C_27_H_26_N_5_. Obtained: 420.2197.

N,9-bis(4-methoxybenzyl)-9H-purin-6-amine **(L4-2a):** White solid, yield 81%. δH (400.45 MHz, CDCl_3_): 8.44 (1H, s, NC*H*N)*,* 7.65 (1H, s, NC*H*N)*,* 7.30 (2H, d, NCH_2_*Ph,H-orto-orto´*, J=8.80 Hz), 7.24 (2H, d, NHCH_2_*Ph*, *H-orto-orto´* J=8.80 Hz), 6.87 (2H, d, NCH_2_*Ph*, *H-meta-meta´* J=6.80 Hz), 6.85 (2H, d, NHCH_2_*Ph*, *H-meta-meta´* J=6.80 Hz), 6.25 (1H, bs, N*H*), 5.28 (2H, s, NC*H_2_*Ph ), 4.81 (2H, s, NHC*H_2_*Ph), 3.78 (6H, s, OC*H_3_* x2). δC (100.70 MHz, CDCl_3_): 167.89, 159.83, 158.22, 139.86, 132.60, 131.01, 130.47, 129.53, 129.29, 128.94, 114.57, 114.22, 68.30, 55.43, 46.95, 38.88. ES+HRMS: Calculated M+H=376.1774 C_21_H_22_N_5_O_2_. Obtained: 376.1753.

N,9-bis(4-methoxybenzyl)-8-methyl-9H-purin-6-amine **(L4-2b):** White solid, yield 44%. δH (400.45 MHz, CDCl_3_): 8.40 (1H, s, NC*H*N)*,* 7.28 (2H, d, NCH_2_*Ph,H-orto-orto´*, J=8.41 Hz), 7.10 (2H, d, NHCH_2_*Ph*, *H-orto-orto´* J=8.81 Hz), 6.82 (2H, d, NCH_2_*Ph*, *H-meta-meta´* J=7.60 Hz), 6.80 (2H, d, NHCH_2_*Ph*, *H-meta-meta´* J=8.41 Hz), 6.28 (1H, bs, N*H*), 5.26 (2H, s, NC*H_2_*Ph ), 4.78 (2H, s, NHC*H_2_*Ph), 3.76 (3H, s, OC*H_3_*), 3.75 (3H, s, OC*H_3_*), 2.41 (3H, s, CC*H_3_*). δC (100.70 MHz, CDCl_3_): 159.42, 159.02, 153.62, 152.53, 150.70, 149.03, 130.71, 129.22, 128.81, 128.59, 118.41, 55.36, 45.45, 14.37. ES+HRMS: Calculated M+H=390.1930 C_22_H_24_N_5_O_2_. Obtained: 390.1937.

N,9-bis(4-methoxybenzyl)-8-phenyl-9H-purin-6-amine **(L4-2c):** White solid, yield 65%. δH (400.45 MHz, CDCl_3_): 8.46 (1H, s, NC*H*N)*,* 7.58 (2H, d, -*Ph*, J=6.80 Hz), 7.46 (3H, m, -*Ph),*  7.34 (2H, d, NCH_2_*Ph,H-orto-orto´*, J=8.41 Hz), 7.00 (2H, d, NHCH_2_*Ph*, *H-meta-meta´* J=8.41 Hz), 6.86 (2H, d, NCH_2_*Ph*, *H-orto-orto´* J=8.81 Hz), 6.80 (2H, d, NHCH_2_*Ph*, *H-meta-meta´* J=8.81 Hz), 6.20 (1H, bs, N*H*), 5.41 (2H, s, NC*H_2_*Ph ), 4.84 (2H, s, NHC*H_2_*Ph), 3.79 (3H, s, OC*H_3_*), 3.76 (3H, s, OC*H_3_*). ). δC (100.70 MHz, CDCl_3_): 159.35, 159.26, 153.13, 139.66, 130.39, 129.72, 129.43, 129.28, 129.00, 128.32, 114.36, 114.24, 55.44, 55.39, 47.12, 46.67. ES+HRMS: Calculated M+H=452.2087 C_27_H_26_N_5_O_2_. Obtained: 452.2054.

- **N,9-diisopropyl-9H-purin-6-amine (L4-3a):** White solid, yield 56%, bp: 115˚C. δH (400.45 MHz, CDCl_3_): 8.35 (1H, s, NC*H*N)*,* 7.80 (1H, s, NC*H*N)*,* 5.70 ((1H, bs, N*H*), 4.80 (1H, m, C*H*(CH_3_)_2_), 4.56 (1H, m, C*H*(CH_3_)_2_), 1.59 (6H, d, J= 6.9, -CH(C*H_3_*)_2_), 1.31 (6H, d, J= 6.5, -CH(C*H_3_*)_2_). δC (100.70 MHz, CDCl_3_): 152.54, 137.39, 119.97, 47.13, 43.01, 23.20, 22.85. ES+HRMS: Calculated M+H=220.1562 C_11_H_18_N_5_. Obtained: 220.1559.
- **N,9-diisopropyl-8-methyl-9H-purin-6-amine (L4-3b):** Yellow solid, yield 30%. δH (400.45 MHz, CDCl_3_): 8.30 (1H, s, NC*H*N)*,* 4.70 (1H, m, C*H*(CH_3_)_2_), 4.54 ((1H, bs, N*H*), 4.21 (1H, m, C*H*(CH_3_)_2_), 2.58 (3H, s, C*H_3_*), 1.65 (6H, d, J= 6.9, -CH(C*H_3_*)_2_), 1.31 (6H, d, J= 6.5, -CH(C*H_3_*)_2_). δC (100.70 MHz, CDCl_3_): 152.22, 148.30, 132.60, 131.00, 118.46, 48.26, 38.88, 22.18, 21.43, 15.22. ES+HRMS: Calculated M+H=234.1719 C_12_H_20_N_5_. Obtained: 234.1720.
- **N,9-diisopropyl-8-phenyl-9H-purin-6-amine (L4-3c):** Yellow solid, yield 30%. δH (400.45 MHz, CDCl_3_): 8.38 (1H, s, NC*H*N)*,* 7.60-7.57 (2H, m, Ph), 7.55-7.52 (3H, 4.70 (1H, m, C*H*(CH_3_)_2_), 5.75 ((1H, bs, N*H*), 4.68 (1H, m, C*H*(CH_3_)_2_), 4.56 (1H, m, C*H*(CH_3_)_2_), 1.70 (6H, d, J= 6.9, -CH(C*H_3_*)_2_), 1.33 (6H, d, J= 6.5, -CH(C*H_3_*)_2_). δC (100.70 MHz, CDCl_3_): 151.16, 130.20, 129.48, 129.04, 49.77, 28.84, 23.18, 21.51. ES+HRMS: Calculated M+H=296.1875 C_17_H_22_N_5_. Obtained: 296.1893.
- **N,9-bis(4-chlorobenzyl)-9H-purin-6-amine (L4-4a):** White solid, yield 35%, bp:135˚C. δH (400.45 MHz, CDCl_3_): 8.37 (1H, s, NC*H*N)*,* 7.79 (1H, s, NC*H*N)*,* 7.30 (2H, d, J=6.8, Ph)*,* 7.28 (2H, d, J=4.3, Ph)*,* 7.26 (2H, d, J=4.3, Ph)*,* 7.24 (2H, d, J=8.5, Ph)*,* 6.49 ((1H, bs, N*H*), 5.35 (2H, s, C*H*_2_), 4.87 (2H, s, C*H*_2_). δC (100.70 MHz, CDCl_3_): 153.60, 149.06, 137.24, 134.19, 131.31, 129.31, 129.17, 128.86, 129.57, 118.47, 45.37, 14.37. ES+HRMS: Calculated M+H=384.0783 C_19_H_16_N_5_Cl_2_. Obtained: 384.0758.
- **N,9-bis(4-chlorobenzyl)-8-methyl-9H-purin-6-amine (L4-4b):** White solid, yield 10%, bp:81˚C. δH (400.45 MHz, CDCl_3_): 8.38 (1H, s, NC*H*N)*,* 7.36 (2H, d, J=7.44, Ph)*,* 7.33 (2H, d, Ph)*,* 7.28 (2H, d, J=7.1, Ph)*,* 7.24 (2H, d, J=6.8, Ph)*,* 6.2 ((1H, bs, N*H*), 5.31 (2H, s, C*H*_2_), 4.84 (2H, s, C*H*_2_), 2.43 (3H, s, C*H_3_*). δC (100.70 MHz, CDCl_3_): 132.58, 131.02, 129.51, 129.27, 128.95, 118.47, 46.95, 38.86. ES+HRMS: Calculated M+H=398.0939 C_20_H_18_N_5_Cl_2_. Obtained: 398.0936.
- **N,9-bis(4-chlorobenzyl)-8-phenyl-9H-purin-6-amine (L4-4c):** Yellow oil, yield 30%, bp:100˚C. δH (400.45 MHz, CDCl_3_): 8.43 (1H, s, NC*H*N)*,* 7.56-7.46 (m, 5H, Ph), 7.35 (2H, d, J=8.5, Ph)*,* 7.30 (2H, d, Ph)*,* 7.24 (2H, d, J=8.8, Ph)*,* 7.0 (2H, d, J=8.4, Ph), 6.39 ((1H, bs, N*H*), 5.43 (2H, s, C*H*_2_), 4.9 (2H, s, C*H*_2_). δC (100.70 MHz, CDCl_3_): 155.30, 146.16, 134.05, 129.30, 129.26, 129.16, 129.15, 128.96, 128.35, 119.29, 46.60. ES+HRMS: Calculated: 460.1096 M+H= C_25_H_20_N_5_Cl_2_. Obtained: 460.1076.

**N,9-bis(4-bromophenethyl)-9H-purin-6-amine (L4-7a):** Yellow solid, yield 28%. δH (400.45 MHz, CDCl_3_): 10.40 (1H, s, N*H*), 8.71 (1H, s, NC*H*N)*,* 7.64 (1H, s, NC*H*N)*,* 7.41 (2H, d, NCH*_2_*CH_2_*Ph,H-orto-orto´*, J=6.7 Hz), 7.38 (2H, d, NHCH*_2_*CH*_2_Ph*, *H-orto-orto´* J=6.7 Hz), 7.20 (2H, d, NCH_2_CH_2_*Ph*, *H-orto-orto´* J=6.7 Hz), 6.95 (2H, d, NHCH_2_CH_2_Ph, *H-meta-meta´* J=6.7 Hz), 4.50 (2H, t, J=5.48, NC*H_2_*CH_2_Ph), 4.47 (2H, dt, J=6.32, NHC*H_2_*CH_2_Ph), 3.18 (2H, t, J=5.60, NCH_2_C*H_2_*Ph), 2.96 (2H, t, J=6.30, NHCH_2_C*H_2_*Ph). δC (100.70 MHz, CDCl_3_): 164.99, 152.16, 151.20, 137.87, 136.13, 142.74, 132.21, 131.56, 130.98, 130.52, 122.50, 121.38, 45.51, 42.17, 35.58, 33.82. ES+HRMS: Calculated M+H=500.0085 C_21_H_20_N_5_Br_2_. Obtained: 500.0118.

**N,9-bis(4-bromophenethyl)-8-phenyl-9H-purin-6-amine (L4-7c):** Yellow solid, yield 61%. δH (400.45 MHz, CDCl_3_): 8.44 (1H, s, NC*H*N)*,* 7.50-7.43 (3H, m, Ph), 7.38 (2H, d, N CH_2_CH_2_*Ph,H-orto-orto´*, J=6.60 Hz), 7.36-7.34 (2H, m, Ph), 7.23 (2H, d, NH CH_2_CH_2_*Ph*, *H-orto-orto´* J=6.72 Hz), 7.13 (2H, d, N CH_2_CH_2_*Ph*, *H-meta-meta´* J=6.6 Hz), 6.70 (2H, d, NH CH_2_CH_2_*Ph*, *H-meta-meta´* J=6.72 Hz), 4.50 (2H, t, J=5.76, NC*H_2_*CH_2_Ph), 3.93 (2H, m, NHC*H_2_*CH_2_Ph), 3.01 (2H, t, J=5.72, NCH_2_C*H_2_*Ph), 2.97 (2H, t, J=5.88, NHCH_2_C*H_2_*Ph). δC (100.70 MHz, CDCl_3_): 153.08, 151.01, 137.99, 136.27, 131.76, 131.70, 130.75, 130.48, 130.19, 129.55, 128.99, 128.90, 120.93, 120.40, 44.99, 35.14. ES+HRMS: Calculated M+H=576.0398 C_27_H_24_N_5_Br_2_. Obtained: 576.0355.

**N,9-bis(3-chlorobenzyl)-9H-purin-6-amine (L4-8a):** White solid, yield 47%, bp:141˚C. δH (400.45 MHz, CDCl_3_): 8.45 (1H, s, NC*H*N)*,* 7.69 (1H, s, NC*H*N)*,* 7.40-7.27 (6H, m, Ph)*,* 7.19-7.17 (2H, m, Ph)*,* 6.97 ((1H, bs, N*H*), 5.35 (2H, s, C*H*_2_), 4.89 (2H, s, C*H*_2_). δC (100.70 MHz, CDCl_3_): 153.60, 149.58, 139.88, 135.12, 134.63, 130.52, 130.07, 128.83, 127.95, 127.83, 127.78, 125.98, 125.86, 119.66, 46.68. ES+HRMS: Calculated M+H=384.0783 C_19_H_16_N_5_Cl_2_. Obtained: 384.0798.

- **N,9-bis(3-chlorobenzyl)-8-methyl-9H-purin-6-amine (L4-8b):** White oil, yield 16%. δH (400.45 MHz, CDCl_3_): 8.39 (1H, s, NC*H*N)*,* 7.49-7.32 (7H, m, Ph)*,* 7.15-7.11 (1H, m, Ph)*,* 6.48 ((1H, bs, N*H*), 5.43 (2H, s, C*H*_2_), 4.98 (2H, s, C*H*_2_), 2.54 (3H, s, C*H_3_*). δC (100.70 MHz, CDCl_3_): 149.03, 140.69, 137.60, 135.00, 134.49, 130.34, 129.91, 128.42, 127.75, 127.61, 127.13, 125.79, 118.30, 45.31, 14.24. ES+HRMS: Calculated M+H=398.0939 C_20_H_18_N_5_Cl_2_. Obtained: 398.0956.
- **N,9-bis(3-chlorobenzyl)-8-phenyl-9H-purin-6-amine (L4-8c):** Yellow oil, yield 50%. δH (400.45 MHz, CDCl_3_): 8.47 (1H, s, NC*H*N)*,* 7.50-7.40 (m, 5H, Ph), 7.33-7.21 (6H, m, Ph)*,* 7.10-6.95 (2H, m, Ph)*,* 6.39 ((1H, bs, N*H*), 5.46 (2H, s, C*H*_2_), 4.90 (2H, s, C*H*_2_). δC (100.70 MHz, CDCl_3_): 151.30, 140.65, 138.40, 135.00, 134.64, 130.59, 130.36, 130.08, 129.13, 128.34, 127.99, 127.82, 127.07, 125.99, 124.98 119.46, 46.60. ES+HRMS: Calculated: 460.1096 M+H= C_25_H_20_N_5_Cl_2_. Obtained: 460.1068.

**N,9-bis(2-chlorobenzyl)-9H-purin-6-amine (L4-9a):** Yellow solid, yield 47%. δH (400.45 MHz, (CD_3_)_2_SO): 8.41 (1H, s, NC*H*N)*,* 8.26 ((1H, bs, N*H*), 8.18 (1H, s, NC*H*N)*,* 7.53-7.03 (8H, m, Ph)*,* 5.49 (2H, s, C*H*_2_), 4.77 (2H, d, C*H*_2_). δC (100.70 MHz, (CD_3_)_2_SO): 152.65, 149.13, 141.24, 133.99, 132.00, 131.73, 129.69, 129.52, 129.38, 129.05, 128.30, 128.03, 127.62, 127.10, 119.07, 44.24. ES+HRMS: Calculated M+H=384.0783 C_19_H_16_N_5_Cl_2_. Obtained: 384.0798.

**N,9-bis(2-chlorobenzyl)-8-methyl-9H-purin-6-amine (L4-9b):** White oil, yield 16%. δH (400.45 MHz, CDCl_3_): 8.41 (1H, s, NC*H*N)*,* 7.56-7.11 (8H, m, Ph)*,* 6.65 ((1H, bs, N*H*), 5.50 (2H, s, C*H*_2_), 5.03 (2H, d, C*H*_2_), 2.47 (3H, s, C*H_3_*). δC (100.70 MHz, CDCl_3_): 153.66, 149.41, 139.10, 133.96, 133.27, 132.49, 129.95, 129.88, 129.69, 129.31, 128.94, 127.57, 127.47, 127.09, 118.58, 45.35, 14.13. ES+HRMS: Calculated M+H=398.0939 C_20_H_18_N_5_Cl_2_. Obtained: 398.0941.

**N,9-bis(2-chlorobenzyl)-8-phenyl-9H-purin-6-amine (L4-9c):** Yellow oil, yield 11%. δH (400.45 MHz, CDCl_3_): 8.43 (1H, s, NC*H*N)*,* 7.58-7.51 (m, 3H, Ph), 7.47-7.37 (6H, m, Ph)*,* 7.26-7.20 (2H, m, Ph)*,* 7.18-7.12 (1H, m, Ph)*,* 6.78-6.71 (1H, df, Ph)*,* 6.43 ((1H, bs, N*H*), 5.55 (2H, s, C*H*_2_), 5.01 (2H, d, C*H*_2_). δC (100.70 MHz, CDCl_3_): 153.35, 151.34, 135.96, 133.94, 132,16, 130.48, 130.08, 129.91, 129.74, 129.16, 129.06, 128.83, 127.53, 127.17, 127.14, 119.60, 45.21. ES+HRMS: Calculated: 460.1096 M+H= C_25_H_20_N_5_Cl_2_. Obtained: 460.1053.
